# Targeting the lipid desaturation network in cancer: from metabolic plasticity to precision therapeutics

**DOI:** 10.1186/s13046-026-03747-x

**Published:** 2026-06-17

**Authors:** Justyna J. Gleba, John A. Copland, Han W. Tun

**Affiliations:** 1 Department of Cancer Biology, Mayo Clinic, Jacksonville, FL USA; 2 Department of Hematology/Oncology, Mayo Clinic, Jacksonville, FL USA

**Keywords:** Lipid desaturation, SCD1, FADS2, Ferroptosis, Metabolic plasticity, Sapienic shunt, MTI-301 (SSI-4), Precision therapeutics, ER stress, Cancer metabolism

## Abstract

Lipid desaturation is a fundamental biochemical process essential for maintaining membrane fluidity, energy storage, and cellular signaling. It is increasingly recognized that this homeostatic network is frequently dysregulated by malignant cells to support proliferation, evade programmed cell death, and facilitate immune evasion. There are two primary lipid desaturation pathways: the conversion of saturated fatty acids (SFAs) to monounsaturated fatty acids (MUFAs) by stearoyl-CoA desaturase 1 (SCD1), and the biosynthesis of long-chain polyunsaturated fatty acids (LC-PUFAs) via the fatty acid desaturases (FADS). This review explores how tumors utilize the SCD1 axis to mitigate lipotoxic endoplasmic reticulum (ER) stress and ferroptosis. Furthermore, we discuss how the FADS axis presents a distinct metabolic paradox: while it promotes oncogenic signaling and structural plasticity, it concurrently creates an actionable vulnerability to ferroptosis by enriching membranes with peroxidation-prone PUFAs. This metabolic rewiring provides a strong biological rationale for precision therapeutics.

We trace the clinical development of desaturase inhibitors, highlighting the recent entry of SCD1 inhibitor, MTI-301, in a Phase 1 clinical trial for solid tumors and the potential repurposing of Aramchol, while detailing how FADS2 plasticity (the “sapienic shunt”) drives therapeutic resistance. By integrating these insights into desaturation lipidomics, metabolic modulation via diet-drug interactions, synergistic combination regimens, and stimuli-responsive nanomedicine, we highlight the translational potential of targeting lipid desaturation to overcome metabolic plasticity and treatment resistance in aggressive malignancies.

## Introduction: lipid desaturation as a metabolic dependency of cancer cells

Cellular membranes are highly dynamic structures that tightly regulate signal transduction, energy homeostasis, and stress adaptation [1, 2]. Central to this process is fatty acid desaturation, the oxygen-dependent biochemical insertion of *cis*-double bonds into hydrocarbon chains [3]. This structural modification transforms rigid saturated fatty acids (SFAs) into flexible unsaturated forms (UFAs), a shift that fundamentally alters membrane biophysics, modulates the conformational states of transmembrane proteins, and gLipid desaturase enzymes, metabolic pathways, and role in cancerenerates the essential precursors for key immune-signaling molecules [4, 5].

Reprogramming of lipid desaturation is a hallmark of malignant transformation [6]. To meet the demands of membrane biogenesis during continuous proliferation, tumors typically upregulate *de novo* lipogenesis, rapidly synthesizing fatty acids from carbohydrates via enzymes like fatty acid synthase (FASN) [7, 8]. However, this heightened anabolic drive creates a major biochemical bottleneck. *De novo* lipogenesis primarily generates fully saturated lipids, such as palmitate [9]. If left unmodified, the accumulation of these rigid SFAs triggers severe endoplasmic reticulum (ER) stress and lipoapoptosis [10, 11]. To survive their own metabolic burden, cancer cells adapt by overexpressing lipid desaturases, primarily SCD1 and FADS2 [12, 13]. By catalyzing conversion of SFAs to MUFAs, desaturases buffer lipotoxicity, suppress anti-tumor immunity, and protect against ferroptosis, an iron-dependent, non-apoptotic cell death driven by unchecked lipid peroxidation [14–16].

The dependency of cancer cells on lipid desaturases creates a metabolic vulnerability. This review provides an overview of the current understanding of lipid desaturation in oncology, outlining the molecular mechanisms of desaturase-driven tumorigenesis and evaluating the therapeutic potential of targeting these pathways in clinical settings.

### The architecture of cellular lipid homeostasis and survival: desaturation, synthesis, remodeling, storage, and redox control

Lipid desaturation occurs primarily in the ER, mediated by an electron-transfer cascade consisting of NADH-cytochrome b5 reductase, cytochrome b5, and terminal iron-containing desaturases [17, 18]. In mammalian cells, this process is regulated by two functionally distinct enzymatic branches that maintain cellular lipid homeostasis (Table 1). Critically, lipid desaturation is deeply integrated with *de novo* lipid synthesis (DNL), phospholipid remodeling via the Lands cycle, neutral lipid storage in lipid droplets, and redox-buffering systems that collectively determine cellular lipid homeostasis and susceptibility to ferroptosis, lipotoxicity, and ER stress related apoptosis.

**Table 1:** Lipid desaturase enzymes, metabolic pathways, and role in cancer

Enzyme	Pathway	Substrates	Products	Role	Adaptation in cancer	References
SCD1	SFA-MUFA axis	Palmitoyl-CoA (16:0), Stearoyl-CoA (18:0)	Palmitoleoyl-CoA (16:1n-7), Oleoyl-CoA (18:1n-9)	Maintains membrane fluidity, regulates lipid droplet biogenesis, and controls energy homeostasis.	Increases membrane plasticity; buffers lipotoxic ER stress; dilutes oxidizable lipids to suppress ferroptosis; stabilizes Wnt/β-catenin stemness signaling.	[11, 19–31]
FADS1	Canonical PUFA network	Intermediate PUFAs	Long-chain PUFAs (e.g., arachidonic acid, EPA, DHA)	Catalyzes the terminal synthesis of complex LC-PUFAs; provides precursors for eicosanoid production.	Promotes pro-tumorigenic inflammation; synthesizes immunosuppressive PGE2 to alter the tumor microenvironment.	[32–37]
FADS2	Canonical PUFA network	Intermediate LC-PUFAs	Linoleic acid (LA), α-linolenic acid (ALA)	Acts as the rate-limiting enzyme in endogenous LC-PUFA biosynthesis from dietary sources.	Enriches membranes with oxidizable bis-allylic PUFAs, establishing a vulnerability to ferroptosis.	[38–48]
FADS2	Sapienic shunt	Palmitate (16:0)	Sapienic acid (cis-6-16:1)	Possesses minor physiological activity, primarily localized to human sebaceous glands.	Engaged during severe hypoxia or SCD1 inhibition; bypasses SCD1 dependence to restore membrane fluidity and mediate resistance to ferroptosis.	[49–53]

#### The SCD1-driven SFA-MUFA axis

Stearoyl-CoA desaturase 1 (SCD1) regulates the balance between saturated and monounsaturated fatty acids (the SFA/MUFA ratio) [19]. As a delta9-desaturase, it catalyzes the insertion of a single *cis*-double bond into stearoyl-CoA (C18:0) and palmitoyl-CoA (C16:0) to generate flexible MUFAs, oleoyl-CoA (C18:1n-9) and palmitoleoyl-CoA (C16:1n-7) [20, 21]. Given its central role in energy homeostasis, SCD1 expression is under tight transcriptional control, coordinated primarily by sterol regulatory element-binding proteins (SREBPs), LXR, and the PPAR family [54, 55].

Beyond maintaining membrane fluidity, SCD1 modulates cellular energy metabolism through several interconnected mechanisms. Crucially, SCD1 provides MUFA substrates for triacylglycerol (TAG) synthesis. Diacylglycerol acyltransferases, DGAT1 and DGAT2, catalyze the terminal step of the synthesis, esterifying diacylglycerol with fatty acyl-CoA to form neutral lipids which are stored in lipid droplets [22, 23]. The substrate specificities of DGAT1 and DGAT2 are not fully resolved and appear to be context- and tissue-dependent [56]. DGAT1 and DGAT2 serve distinct functional roles: DGAT2 preferentially channels fatty acids derived from *de novo* lipogenesis (DNL) into TAG, whereas DGAT1 more broadly processes exogenous fatty acids and lipids recycled through autophagy-mediated lipophagy [56–59]. By funneling lipid overflow into these inert storage pools, SCD1-derived MUFAs serve as a key metabolic sink [24]. This sequestration prevents the harmful accumulation of unesterified saturated lipids and toxic intermediates (such as ceramides and diacylglycerols), thereby protecting the ER from structural rigidification and blocking the mechanosensory auto-activation of UPR sensors such as IRE1-alpha and PERK [11, 25].

The SCD1-AMPK axis serves as a key metabolic regulator. Under nutrient-rich conditions, SCD1 drives lipid biosynthesis. However, during energetic stress, AMPK is activated, phosphorylating and inhibiting acetyl-CoA carboxylase (ACC). This suppresses *de novo* lipogenesis and redirects fatty acids toward oxidation [26, 60]. SCD1 knockout has been shown to result in AMPK activation [26], whereas AMPK activation has been associated with SCD1 downregulation via SREBP1 [61]. Cancer cells frequently rewire these metabolic pathways. For instance, in hepatocellular carcinoma, lactate-driven AMPK activation upregulates SCD1, conferring strong resistance to ferroptosis and promoting tumor cell survival [60]. Furthermore, SCD1 activity is closely linked to autophagy. Inhibiting SCD1 induces lipotoxic ER stress, which in turn triggers compensatory autophagy. In glioblastoma models, combining SCD1 ablation with autophagy inhibition results in synergistic cell death, demonstrating that cancer cells rely on autophagy to survive the metabolic stress of SCD1 blockade [62, 63].

Beyond cancer, SCD1 dysregulation contributes to several systemic diseases. Because SCD1 regulates lipid partitioning in hepatocytes, excessive lipogenesis through this pathway drives metabolic dysfunction-associated steatotic liver disease (MASLD) and steatohepatitis (MASH) [64]. In adipocytes, SCD1 balances triacylglycerol storage with fatty acid oxidation, consequently, *Scd1*-deficient mice are highly resistant to diet-induced obesity [26, 27, 64, 65]. In the cardiovascular system, SCD1-derived oleate modulates cardiomyocyte membrane fluidity and regulates macrophage lipid handling during atherosclerosis [64, 65]. In the central nervous system, while baseline SCD1 activity maintains myelin lipid composition, targeted SCD1 inhibition has emerged as a potential strategy to reduce lipid-driven proteotoxicity in Parkinson’s disease and related synucleinopathies [66]. Because SCD1 plays such broad physiological roles across healthy tissues, systemic inhibition can cause significant on-target toxicities. Therefore, oncological therapies targeting SCD1 will likely require tissue-restricted or conditionally activated delivery systems to achieve a safe therapeutic window.

#### The FADS-driven PUFA network

Because mammalian cells lack the enzymatic capacity to synthesize essential linoleic acid (LA, 18:2n-6) and alpha-linolenic acid (ALA, 18:3n-3) *de novo*, maintaining optimal levels of long-chain polyunsaturated fatty acids (LC-PUFAs) depends on dietary intake paired with an endogenous enzymatic pathway consisting of desaturases and elongases [38]. FADS2 acts as primary rate-limiting enzyme, catalyzing desaturation of LA and ALA [39], while downstream FADS1 (delta5-desaturase) completes the synthesis of complex LC-PUFAs, most notably arachidonic acid (AA, 20:4n-6), eicosapentaenoic acid (EPA, 20:5n-3), and docosahexaenoic acid (DHA, 22:6n-3) [32].

These PUFAs possess significant conformational flexibility. Because they sweep out through a large spatial volume, they are biophysically incompatible with flat, rigid cholesterol molecules [40]. This steric incompatibility drives spontaneous phase separation, promoting the clustering of cholesterol and sphingolipids into dense liquid-ordered microdomains known as lipid rafts, which serve as essential signaling platforms [41, 42].

Furthermore, PUFAs serve as precursors for bioactive signaling molecules [33]. Upon tissue injury, released AA is funneled into the cyclooxygenase (COX) and lipoxygenase (LOX) cascades to produce pro-inflammatory eicosanoids (prostaglandins, leukotrienes) [34, 35]. During the resolution phase of inflammation, competitive substrate shifts allow omega-3 PUFAs (EPA, DHA) to be converted into specialized pro-resolving mediators (SPMs) such as resolvins and protectins, driving the termination of immune responses [36].

After synthesis via the FADS pathway, unesterified PUFAs are sorted into specific subcellular compartments. To prevent lipotoxicity, excess PUFAs are rapidly converted into triacylglycerols (TAGs) and sequestered within lipid droplets. These lipid droplets act as protective reservoirs, regulating the availability of free PUFAs for membrane biogenesis and eicosanoid signaling [67]. Concurrently, the Lands cycle, a continuous deacylation-reacylation salvage pathway driven by phospholipases and lysophospholipid acyltransferases like LPCAT1 and LPCAT3, remodels the acyl-chain composition of membrane phospholipids. This remodeling provides cells with biophysical flexibility, allowing them to adjust their membrane PUFA content in response to metabolic and environmental stress [68].

Ultimately, the subcellular localization of PUFAs dictates their downstream function. For example, the incorporation of PUFAs into plasma membrane phosphatidylethanolamines (PEs) heavily primes cells for ferroptosis. In contrast, PUFAs sequestered in lipid droplets or integrated into mitochondrial cardiolipin serve highly distinct structural and bioenergetic roles [67, 69]. Despite these established pathways, key mechanistic questions remain. Understanding exactly how cells regulate PUFA flux between neutral lipid storage and vulnerable membrane pools, and how tumors co-opt this spatial sorting to survive, remains a major ongoing challenge in cancer metabolism research.

#### The ferroptosis balance

Sensitivity to ferroptosis is governed by a tightly regulated lipid balance. This regulatory framework encompasses antioxidant surveillance networks as well as the subcellular sorting of MUFAs and PUFAs, determining whether these lipids are safely sequestered into neutral stores or incorporated into vulnerable membranes [69, 70]. Importantly, this balance is highly adaptable, exhibiting both lineage-specific and microenvironmental plasticity. Mechanistically, specific PUFAs, predominantly arachidonate (AA) and adrenate (AdA), are conjugated into membrane phosphatidylethanolamines (PEs) by the enzymes ACSL4 and LPCAT3 [43, 69, 71]. Once integrated into the membrane, the bis-allylic carbons of these PE-PUFAs undergo iron-dependent oxidation via Fenton chemistry or lipoxygenase activity. This triggers the accumulation of lipid hydroperoxides, which compromise membrane integrity. Consequently, a cell vulnerability to ferroptosis heavily depends on how PUFAs are trafficked to the membrane [69, 72].

**Fig. 1:**
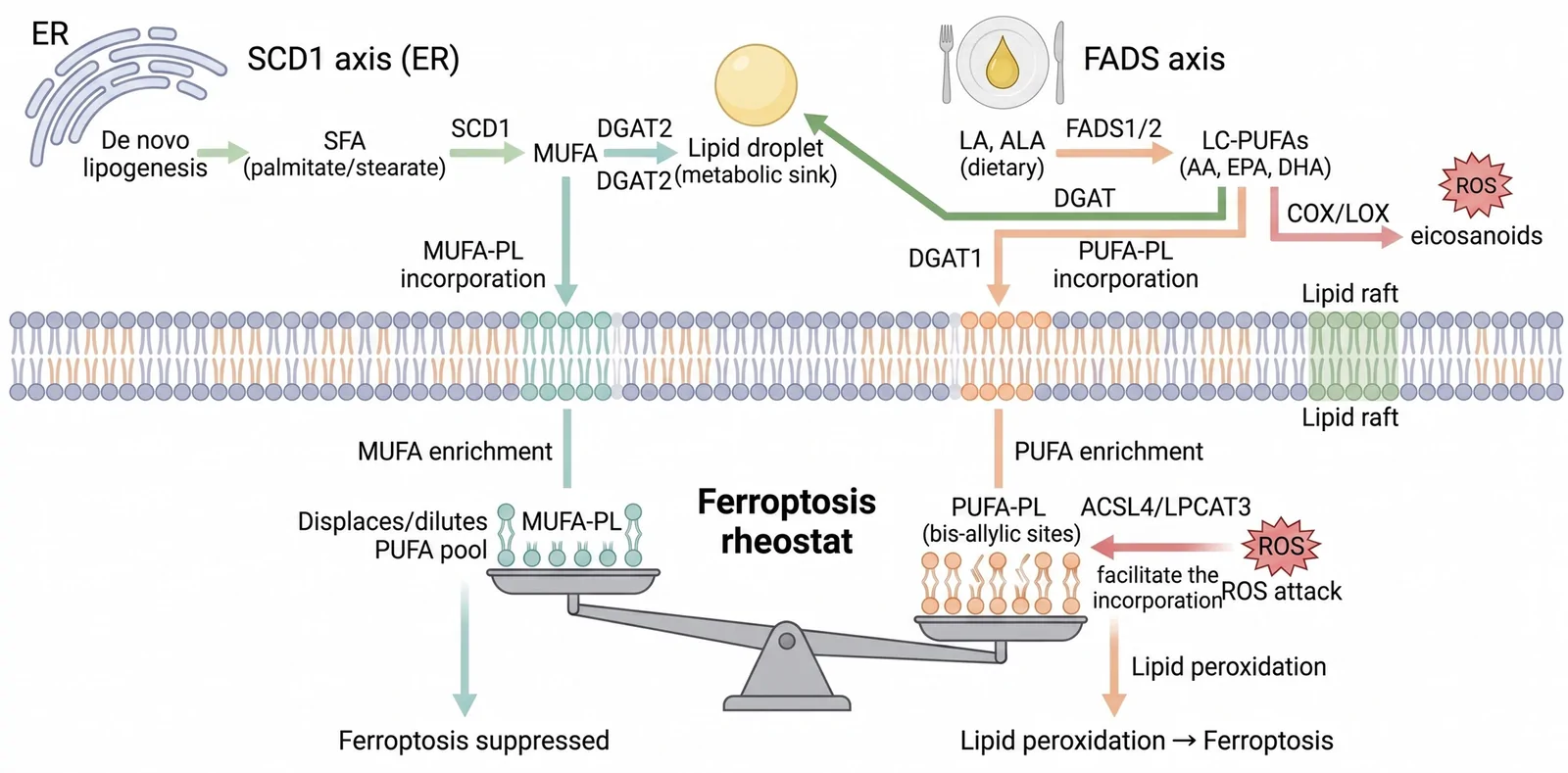
Cellular lipid homeostasis and the ferroptosis balance. Susceptibility to ferroptosis is determined by the relative abundance of membrane monounsaturated fatty acids (MUFAs) versus polyunsaturated fatty acids (PUFAs). In the endoplasmic reticulum (ER), stearoyl-CoA desaturase 1 (SCD1) converts saturated fatty acids (SFAs, primarily palmitate and stearate) derived from de novo lipogenesis into MUFAs. DGAT2 preferentially channels de novo MUFAs into lipid droplets, while DGAT1 processes exogenous and lipophagy-recycled lipids. SCD1-derived MUFAs are incorporated into plasma membrane phospholipids (MUFA-PL), displacing PUFAs to suppress lipid peroxidation and ferroptosis. In parallel, the FADS1/2 axis metabolizes dietary essential fatty acids (LA, ALA) into long-chain PUFAs (LC-PUFAs; AA, EPA, DHA). ACSL4 and LPCAT3 facilitate the incorporation of these LC-PUFAs into the membrane. The bis-allylic sites of PUFA-PLs are susceptible to reactive oxygen species (ROS) attack, resulting in lipid peroxidation and subsequent ferroptotic cell death

DGAT-mediated lipid droplet biogenesis reduces ferroptosis susceptibility by sequestering labile PUFAs into inert TAG pools within the lipid droplet core, effectively depleting the substrate available for lipid peroxidation. This protective storage is important during cell-cycle arrest, as it shields quiescent cells from ferroptosis [73]. However, the duration of this protection depends heavily on the cell’s metabolic state. During catabolic shifts that mobilize lipid droplets via lipophagy, the released polyunsaturated acyl chains are integrated back into the phospholipid bilayer, rapidly restoring sensitivity to ferroptosis [74, 75]. In parallel to this spatial lipid sorting, ferroptosis is regulated by multiple antioxidant networks. Canonical defense systems, including the glutathione peroxidase 4 (GPX4)/glutathione (GSH) axis, the FSP1/CoQ10 oxidoreductase pathway, and the GCH1/BH4 system, which suppresses ferroptosis by driving the production of antioxidant BH4, function as lineage-specific suppressors of lipid peroxidation [76–78] (Fig. 1).

### Metabolic reprogramming: driving the hallmarks of cancer

Across a wide range of solid and hematologic malignancies, desaturase enzymes are frequently overexpressed [79, 80]. This pathological upregulation is not merely a passive consequence of rapid cell division, but an adaptive response that meets the metabolic demands induced by oncogenic growth (Table 2). While lipid desaturases are typically upregulated in cancer, context-dependent downregulation of these enzymes also plays a distinct role in tumor biology, rendering specific malignancies highly susceptible to ferroptosis. For example, in pancreatic cancer models, the nuclear receptor NR4A1 promotes FBXW7-mediated silencing of SCD1, which concurrently drives apoptosis and sensitizes the cells to ferroptosis [81]. Similarly, the transcriptional repressor ATOH8 constitutively blunts SCD1 expression across diverse cancers, including lung adenocarcinoma, colorectal cancer, and hepatocellular carcinoma (HCC), significantly lowering the threshold for ferroptotic cell death in vivo [82]. Within the polyunsaturated fatty acid (PUFA) synthesis pathway, epigenetic silencing of FADS2 by the histone methyltransferase EZH2 markedly increases ferroptosis sensitivity in ovarian carcinoma [83]. Beyond its effects on cell survival, reduced lipid desaturation limits membrane fluidity and impairs the epithelial–mesenchymal transition (EMT), ultimately restricting metastatic dissemination [78, 84].

**Table 2:** Mechanistic impact of lipid desaturation on cancer hallmarks

Cancer hallmark	Cellular stressor	Adaptation	Biological role	References
Proliferation and metastasis	High biophysical demand for rapid membrane expansion and cytoskeletal remodeling.	SCD1 supplies a continuous influx of MUFAs to prevent membrane rigidification.	Facilitates the lateral diffusion of mitogenic receptors (RTKs, GPCRs) and supports invadopodia formation for epithelial-mesenchymal transition.	[85–89]
Cancer stemness	Maintenance of autocrine signaling networks and stem cell self-renewal.	SCD1 generates palmitoleate required for the O-palmitoleoylation of Wnt proteins by Porcupine.	Enables the secretion and oncogenic activation of Wnt ligands, sustaining Wnt/β-catenin pathways.	[28, 29, 90]
Evasion of ER stress	Accumulation of rigid, saturated lipids resulting from elevated de novo lipogenesis.	SCD1 functions as a metabolic sink, converting SFAs into MUFAs for sequestration into inert lipid droplets.	Prevents mechanical auto-activation of UPR apoptotic sensors (IRE1-α, PERK) and subsequent lipoapoptosis.	[10, 11, 24, 25, 91]
Ferroptosis resistance	High oxidative stress and reactive oxygen species accumulation within the TME.	SCD1-derived MUFAs outcompete reactive PUFAs during membrane phospholipid assembly.	Structurally dilutes the pool of oxidizable bis-allylic sites, shielding primary tumors and circulating tumor cells from lipid peroxidation.	[14–16, 30, 31, 74, 92, 93]
Chemoresistance	Prolonged exposure to cytotoxic, lipophilic therapeutics (e.g., paclitaxel, doxorubicin).	Desaturase activity increases the expansion of intracellular lipid droplets.	Dense hydrophobic cores sequester chemotherapeutics away from their intracellular targets, promoting a drug-tolerant persister state.	[61, 94]
Immune evasion	Anti-tumor surveillance mediated by cross-presenting cDC1s and CD8 + T cells.	Wnt-driven repression of CCL4 transcription; extracellular release of MUFAs, oxidized lipids, and PGE2.	Impairs cDC1 recruitment; polarizes TAMs to a suppressive M2-like phenotype; induces CD36-mediated lipotoxic exhaustion in CD8 + T cells.	[37, 95–102]

#### Membrane biophysics: proliferation, invasion, and stemness

Unrestricted cellular proliferation requires rapid membrane expansion. To successfully double its surface area without rigidifying the plasma membrane, a cancer cell requires a substantial, continuous influx of newly synthesized MUFAs [85]. SCD1 introduces the essential structural flexibility that allows mitogenic surface receptors, such as receptor tyrosine kinases (RTKs) and G-protein-coupled receptors (GPCRs), to rapidly diffuse laterally, dimerize, and initiate the intracellular signaling cascades (such as PI3K/AKT/mTOR pathway) that drive the cell cycle [86]. When SCD1 is inhibited, the resulting membrane stiffening restricts the lateral mobility of these receptors and frequently forces the cell into a G1/S phase arrest or severe mitotic failure [87].

This MUFA-driven membrane plasticity is equally vital for metastasis [88]. To escape the primary tumor, carcinoma cells must undergo the epithelial-mesenchymal transition (EMT). Extruding dynamic, actin-driven membrane protrusions (invadopodia) to invade the dense extracellular matrix requires high biophysical fluidity [89]. Furthermore, lipidomic reprogramming is particularly pronounced in cancer stem cells (CSCs) [90]. CSCs maintain a highly specialized, MUFA-skewed lipidome that structurally anchors key stemness receptors (Notch, Hedgehog) and mechanosensory pathways (Hippo/YAP). The secretion and oncogenic activation of Wnt ligands strictly require a specific lipid modification-*O*-palmitoleoylation by the enzyme Porcupine [28]. SCD1 acts as the direct biochemical bridge enabling Wnt/beta-catenin signaling and CSC self-renewal by synthesizing the palmitoleate required by Porcupine [29].

#### Evading ER stress, chemotoxicity, and ferroptosis

As tumors upregulate lipogenesis, they generate substantial quantities of fully saturated palmitate. Cancer cells rely on SCD1 to evade distinct, lipid-driven cell death pathways:

1. Evasion of lipotoxic ER stress: If left unmodified, straight-chain SFAs mechanically stiffen the ER membrane, directly auto-activating transmembrane stress sensors (IRE1-alpha and PERK) to trigger UPR- mediated apoptosis [11, 25]. By converting these toxic SFAs into flexible MUFAs that are safely sequestered into lipid droplets, SCD1 acts as critical metabolic sink, protecting the cell from lipotoxic apoptosis [91].
2. Chemotherapeutic sequestration: When tumors are exposed to cytotoxic treatments, a fraction of cells evades death by entering a resilient drug-tolerant persisted state [94]. The dense hydrophobic cores of expanding lipid droplets act as intracellular sinks, sequestering lipophilic chemotherapeutics (such as paclitaxel and doxorubicin) away from their intended intracellular targets [61].
3. Suppression of ferroptosis: In the highly oxidative tumor microenvironment (TME), cancer cells face a high risk of ferroptosis. SCD1 mitigates this vulnerability through competitive lipid remodeling. By enriching the intracellular pool with peroxidation-resistant MUFAs, SCD1 structurally dilutes the oxidizable PUFA pool, suppressing lipid peroxidation [30, 31]. This resistance is especially critical for circulating tumor cells (CTCs). During hematogenous transit, CTCs face severe oxidative stress in the oxygen-rich bloodstream. SCD1-derived MUFAs provide the biophysical adaptation required to survive circulation and seed distant metastases [92, 93].

#### Immunomodulation: establishing a permissive microenvironment

The metabolic influence of the lipid desaturation network promotes an immunosuppressive TME [95]. Within the tumor cells, aberrant Wnt/beta-catenin signaling, structurally stabilized by SCD1, acts as a transcriptional repressor of CCL4 [96, 97]. This chemokine is an essential chemoattractant required to recruit cross-presenting conventional type 1 dendritic cells (cDC1s). By silencing CCL4, the tumor impairs immune cell recruitment, resulting in an immunologically “cold” or “immune desert” phenotype associated with resistance to immune checkpoint blockade [98].

**Fig. 2:**
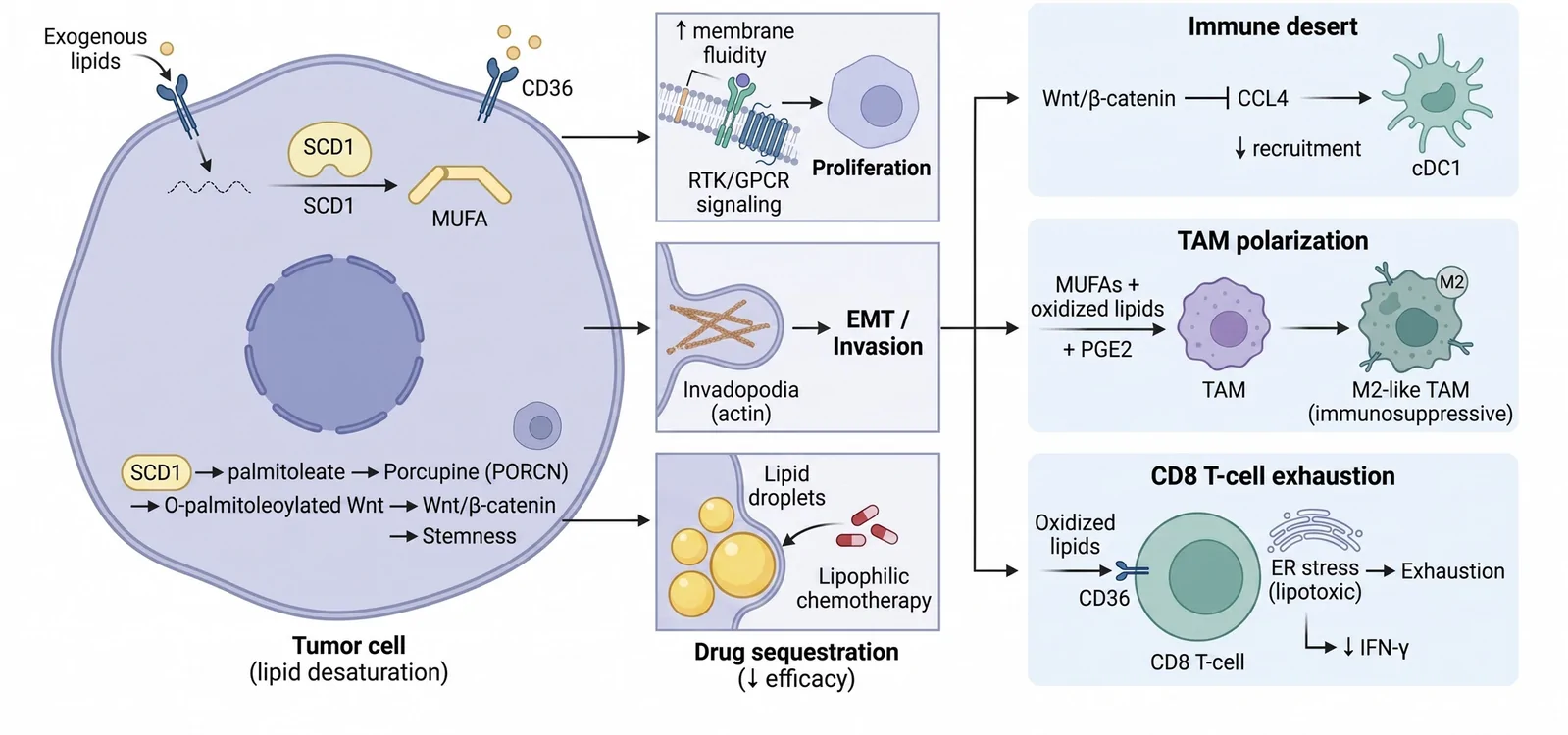
Lipid reprogramming drives cancer hallmarks and TME immunosuppression. Tumor cell lipid desaturation via SCD1 promotes intrinsic malignant progression and microenvironmental immune evasion. Increased MUFA content enhances membrane fluidity, facilitating RTK/GPCR signaling for proliferation and invadopodia formation for epithelial-mesenchymal transition (EMT) and invasion. Tumor cells also use the CD36 receptor to scavenge exogenous lipids, further expanding lipid droplets and altering ferroptosis sensitivity. Palmitoleate generation supports Porcupine (PORCN)-mediated O-palmitoleoylation of Wnt ligands, maintaining cancer stemness through Wnt/beta-catenin signaling. Intracellular lipid droplets sequester lipophilic chemotherapeutic agents, reducing drug efficacy. Within the tumor microenvironment (TME), Wnt/beta-catenin signaling represses CCL4, decreasing cDC1 dendritic cell recruitment to form subsequent ferroptotic cell deathSECTION2Metabolican immune desert. Tumor-derived oxidized lipids and PGE2 polarize tumor-associated macrophages (TAMs) into an immunosuppressive M2-like state. Scavenged oxidized lipids are taken up by CD8+ T cells via the CD36 receptor, inducing lipotoxic ER stress that drives T-cell exhaustion and decreases IFN-gamma production

Within the TME, immune cells experience severe metabolic stress. Tumors release excess MUFAs, oxidized lipids, and FADS1-derived prostaglandin E2 (PGE2) into the stroma [37]. Resident tumor-associated macrophages (TAMs) scavenge these lipids, polarizing toward an immunosuppressive “M2-like” state [99]. Concurrently, infiltrating CD8 + T cells accumulate excess extracellular lipids via surface scavengers such as CD36 [100]. This lipid overload triggers lipotoxic ER stress and lipid peroxidation within the lymphocytes. Consequently, this metabolic burden drives T cells into exhaustion, impairing their ability to secrete interferon-gamma (IFN-gamma) and mediate anti-tumor immunity [101, 102] (Fig. 2).

### Metabolic plasticity and adaptive resistance

While SCD1 provides a broad cytoprotection shield, cancer cells exhibit extensive plasticity to ensure their survival under severe therapeutic or environmental duress.

#### The sapienic shunt: FADS2 as a compensatory bypass

When canonical SCD1 activity is compromised, either by severe hypoxia or targeted pharmacological inhibition, highly aggressive tumors engage an alternative metabolic pathway known as the “sapienic shunt” [49].

Under these stressful conditions, the catalytic flexibility of FADS2 shifts its substrate preference. Rather than desaturating its canonical PUFA substrates, FADS2 directly desaturates palmitate (C16:0) into an atypical MUFA known as sapienic acid (*cis*-6-C16:1) [49, 50]. Because sapienate and its elongated derivatives serve as structural analogs of canonical SCD1 products, they readily incorporate into the plasma membrane. This bypass allows cancer cells to circumvent SCD1 dependence. Sapienate production restores membrane fluidity, mitigates ER stress, and suppresses ferroptosis [51]. Consequently, FADS2 overexpression is a frequent feature of aggressive, therapy-resistant carcinomas, including triple-negative breast, lung, liver, and ovarian cancers [52, 53]. The catalytic plasticity of FADS2, specifically its capacity to shift between different fatty acyl substrates, is regulated by oncogenic and epigenetic signals. In many cancers, mTORC1-SREBP1 signaling acts as a primary transcriptional driver of FADS2 expression. Aberrant mTORC1 activation, whether via PI3K-AKT hyperactivation or loss of the TSC1-TSC2 tumor suppressor complex, significantly upregulates FADS2 and redirects its activity toward sapienate biosynthesis providing MUFAs shield against ferroptosis, even in the absence of concurrent SCD1 blockade [103]. Conversely, the histone methyltransferase EZH2 suppresses FADS2 transcription in ovarian carcinoma resulting in reduced PUFA synthesis, thereby reducing susceptibility to ferroptosis; however, pharmacological EZH2 inhibition effectively restores this sensitivity [83]. Ultimately, the diversion of FADS2 away from the canonical desaturation of PUFA precursors toward sapienate synthesis is not dictated by fixed biochemical properties of the enzyme. Instead, this substrate flexibility acts as an adaptive response shaped by local substrate availability, overall desaturase abundance, and the cellular lipidome.

#### The ferroptosis paradox: priming tumors for oxidative vulnerability

While the FADS network provides structural plasticity and fuels pro-tumorigenic inflammatory signaling, it imposes a significant metabolic trade-off. The sustained synthesis of LC-PUFAs via FADS1/2 incurs a substantial biological cost [44].

**Fig. 3:**
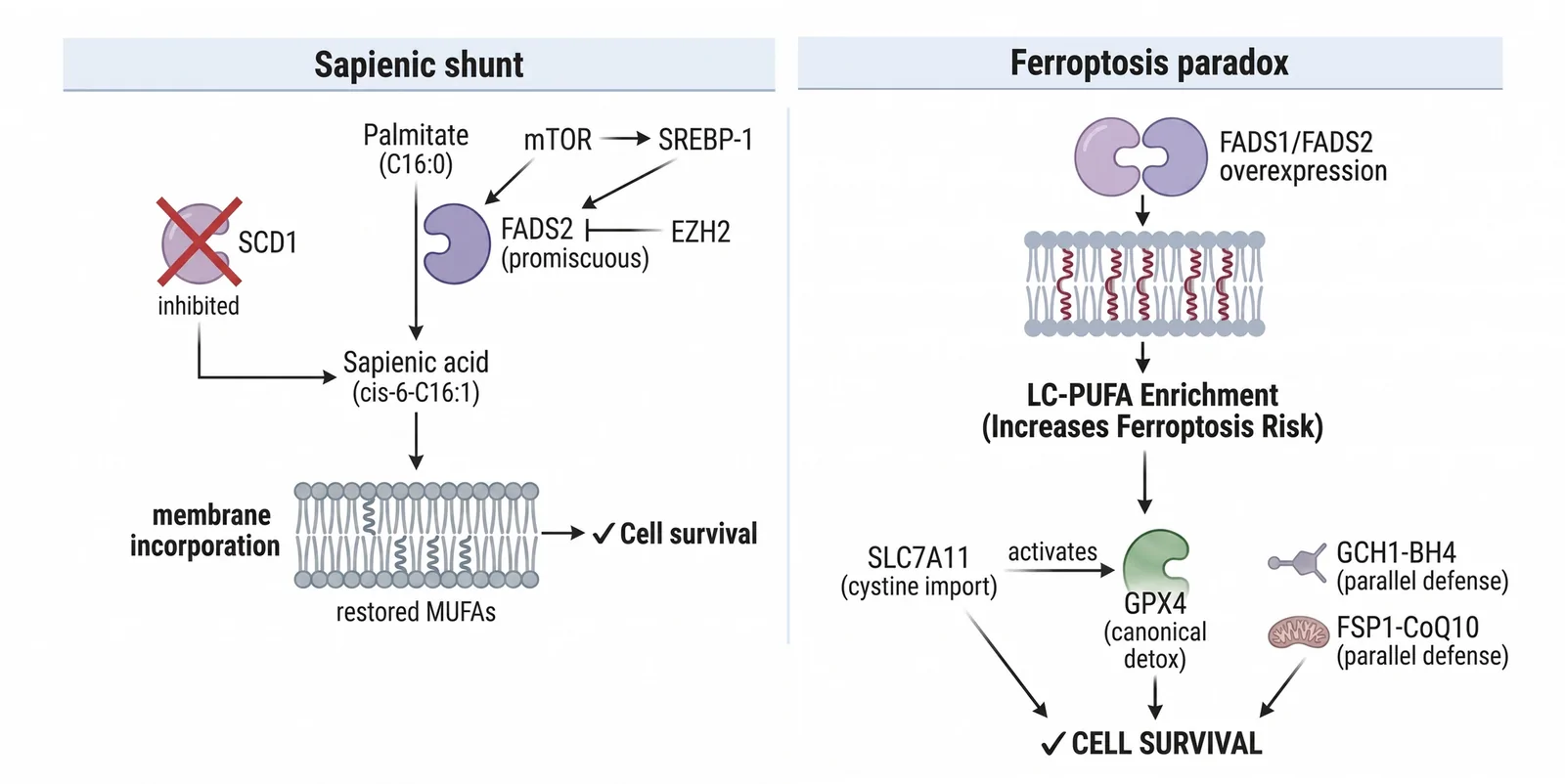
Metabolic plasticity - the sapienic shunt and the ferroptosis paradox. Tumor cells utilize alternate lipid desaturation pathways to maintain homeostasis during targeted therapeutic intervention. Inhibition of SCD1 triggers the sapienic shunt, where the enzyme FADS2 desaturates palmitate (C16:0) into sapienic acid (cis-6-C16:1). Incorporation of this alternative MUFA into the plasma membrane bypasses the SCD1 blockade and restores cell survival. The ferroptosis paradox occurs in tumors with high FADS1/FADS2 expression, leading to membrane LC-PUFA enrichment. While supporting specific malignant traits, elevated membrane LC-PUFA content increases susceptibility to ROS-mediated lipid peroxidation (Lipid-OOH) and basal ferroptosis risk. To counteract this pro-oxidant state, these tumors develop a dependency on antioxidant defense mechanisms, specifically cystine import via SLC7A11 and lipid peroxide detoxification by GPX4. The sapienic shunt is driven by mTOR/SREBP-1 and repressed by EZH2. To survive PUFA-induced oxidative stress, tumors rely on parallel antioxidant defenses (FSP1-CoQ10 and GCH1-BH4) alongside canonical GPX4

Once synthesized, these long-chain PUFAs are enzymatically activated by ACSL4 and incorporated into membrane phospholipids [45]. Because LC-PUFAs contain multiple reactive *bis*-allylic methylene groups, enriching the plasma membrane with these lipids renders the cell highly susceptible to lipid peroxidation [46, 73]. This creates a critical metabolic paradox: while FADS overexpression drives an aggressive metastatic phenotype, it also primes the tumors for ferroptotic cell death [47]. To survive this inherent oxidative vulnerability, these tumors become highly dependent on intracellular antioxidant defense systems, specifically the cystine/glutamate antiporter SLC7A11 and the lipid peroxidase GPX4 [104, 105]. The impact of FADS2 plasticity on ferroptosis susceptibility varies significantly depending on the cell lineage and the tumor microenvironment. In triple-negative breast cancer (TNBC), FADS2 overexpression fuels canonical flux through the FADS pathway, enriching membranes with PUFAs and increasing sensitivity to ferroptosis [106]. Conversely, in urothelial carcinoma, aberrant FADS2 overexpression allows cells to evade ferroptosis by shifting toward non-canonical desaturation [107]. This functional duality is further illustrated by ovarian carcinoma cells in malignant ascites. Within this lipid-rich metastatic niche, SCD1 and FADS2 act as opposing metabolic regulators: SCD1-mediated MUFA enrichment suppresses ferroptosis, whereas continuous FADS2-driven PUFA biosynthesis promotes it [108]. Finally, in spatially restricted, acidic tumor microenvironments, cancer cells upregulate FADS activity to maintain membrane biogenesis. This adaptive rewiring demonstrates how local physicochemical stressors determine the spatial distribution of lipid desaturation within the tumor [109] (Fig. 3).

### Therapeutic translation: from vulnerability to precision oncology

Translating the lipid metabolic dependencies of cancer cells into viable clinical therapies represents a promising frontier in precision oncology. While healthy tissues maintain metabolic flexibility, readily adapting to transient desaturase inhibition by scavenging circulating dietary lipids, poorly vascularized solid tumors often lack this capacity, providing a distinct therapeutic window [110, 111].

#### The evolution and clinical development of SCD1 inhibitors

First-generation systemic SCD1 inhibitors (e.g., A939572, MF-438, CAY10566) demonstrated significant pre-clinical anti-tumor efficacy across diverse xenograft models [19, 112]. These agents induced cell death primarily through a combination of ER stress-mediated apoptosis and ferroptosis [31, 86]. However, the therapeutic window proved narrow. Because healthy sebaceous and meibomian glands rely on SCD1-derived MUFAs to produce sebum and lubricating tear films, chronic inhibition triggered severe dose-limiting on-target toxicities, including dermatitis, alopecia, and dry eye syndrome [113, 114].

To overcome these limitations, recent pharmacological efforts have focused on tissue-selective precision. Researchers engineered brain-penetrant scaffolds (e.g., YTX-7739) specifically for neuro-oncology [66], alongside tumor-directed prodrugs bioactivated by cytochrome P450 isoforms enriched in the TME. These prodrug strategies aim to restrict target engagement primarily to the tumor bed, thereby minimizing toxicity to healthy peripheral tissues [115]. There have been two major clinical milestones:

- MTI-301 (formerly SSI-4): MTI-301 is the first stearoyl-CoA desaturase-1 (SCD1) inhibitor to reach first-in-human clinical trials. It was initially identified using an accelerated computational drug design platform aimed at yielding a highly specific, orally bioavailable compound [116]. The drug is currently undergoing a Phase I dose-escalation and expansion study in patients with advanced solid tumors (NCT06911008). Preclinical studies demonstrate that MTI-301 targets the lipotoxic and oxidative vulnerabilities of cancer cells by inducing endoplasmic reticulum (ER) stress and apoptosis. MTI-301 acts synergistically with standard chemotherapy in acute myeloid leukemia models [117]. Leveraging the high target affinity of the base molecule, MTI-301 was also radiolabeled with Carbon-11 ([11 C]SSI-4) to develop a PET imaging tracer capable of non-invasively mapping SCD1-overexpressing tumors in vivo [118]. In HCC models, the compound promotes ER stress-induced differentiation of cancer stem cells, a mechanism observed to prevent and reverse acquired TKI resistance [119].
- Aramchol: A liver-directed partial SCD1 modulator initially developed for nonalcoholic steatohepatitis (NASH), Aramchol demonstrated strong target engagement and a favorable long-term safety profile in the 52-week Phase 2b ARREST trial [120, 121]. Recent preclinical data indicate that Aramchol synergizes with multi-kinase inhibitors, such as regorafenib, to selectively induce autophagy-mediated cell death in gastrointestinal tumor cells [122]. These findings highlight the clinical viability of partial SCD1 modulation and support the potential repurposing of Aramchol.

#### Targeting the FADS axis and dual-desaturase blockade

Highly plastic tumors frequently engage the FADS2-driven sapienic shunt to evade single-agent SCD1 blockade. Transcriptomic profiling of high-grade ovarian and aggressive solid tumors has revealed a ‘dual-high’ desaturase expression profile strongly correlated with EMT phenotypes and chemoresistance [52, 53].

Accordingly, simultaneous pharmacological co-targeting of both enzymes has emerged as a promising strategy to circumvent this compensatory mechanism [123]. Dual SCD1/FADS2 inhibition induces lipotoxicity. Depleted of canonical and atypical MUFAs and burdened by accumulating SFAs, cancer cells undergo severe ER stress and ferroptosis [124]. In addition, the resulting rigidification of the plasma membrane impairs the function of ATP-binding cassette (ABC) efflux transporters, reversing multidrug resistance and synergizing with platinum-based chemotherapies in recalcitrant tumors [123, 125].

Furthermore, selective FADS inhibition elicits distinct therapeutic effects. FADS1 blockade suppresses the secretion of inflammatory eicosanoids and induces the PERK-eIF2-alpha-ATF4 ER stress axis, driving cell-cycle arrest [126]. Conversely, selective FADS2 inhibition compromises tumor antioxidant defenses, sensitizing these tumors to ferroptosis [127]. (Table 3)

**Table 3:** Pharmacological targeting of lipid desaturase and clinical translation

Agent or class	Target	Stage of development	Mechanism of action and efficacy	Translational limitations and highlights	References
A939572, MF-438, CAY10566	Systemic SCD1	Preclinical (1st generation)	Induces severe ER stress-mediated apoptosis and ferroptosis across diverse cancer models.	Restricted by a narrow therapeutic window; causes dose-limiting toxicities in healthy sebaceous and meibomian glands (alopecia, dermatitis, dry eye).	[19, 31, 112–114]
YTX-7739 and TME-activated prodrugs	Tissue-selective SCD1	Preclinical	Utilizes scaffolds engineered to cross the blood-brain barrier (YTX-7739) or undergo bioactivation via TME-enriched cytochrome P450 isoforms.	Designed to overcome peripheral glandular toxicities by restricting target engagement to the brain or tumor bed.	[66, 115]
MTI-301 (SSI-4)	SCD1	Phase 1 Trial (NCT06911008)	First-in-class systemic inhibitor. Synergizes with standard chemotherapy (AML) and reverses acquired TKI resistance via ER-stress differentiation.	Development is paired with the [11 C]SSI-4 PET imaging tracer for non-invasive in vivo mapping.	[116–119]
Aramchol	Partial SCD1 modulator	Phase 2b (NASH) / Preclinical (Oncology)	Liver-directed partial modulator. Demonstrates synergy with multi-kinase inhibitors (e.g., regorafenib) to trigger autophagy-mediated cell death.	Presents strong repurposing potential due to an established, favorable long-term safety profile from non-oncology clinical trials.	[120–122]
Dual SCD1/FADS2 blockade	SCD1 and FADS2	Preclinical	Simultaneous inhibition depletes both canonical MUFAs and atypical sapienate, inducing lipotoxicity and membrane rigidification.	Required to circumvent the FADS2-driven “sapienic shunt”; successfully impairs ABC efflux transporters to reverse multidrug resistance.	[52, 53, 123–125]
Selective FADS inhibitors	FADS1 or FADS2	Preclinical	FADS1 blockade arrests the cell cycle via the PERK-eIF2α-ATF4 axis. Selective FADS2 inhibition compromises tumor antioxidant capacity.	Generates distinct mechanistic outcomes depending on the targeted isoform; allows for strict sensitization to ferroptosis induction.	[126, 127]

### Emerging paradigms and future directions

The successful clinical translation of lipid desaturation-targeted therapies requires refined pharmacological strategies to maximize therapeutic benefit while minimizing systemic toxicity.

#### Subcellular compartmentalization beyond the ER

Lipid desaturation was traditionally thought to be confined to the ER. However, recent organelle-resolved lipidomics reveal that desaturase biology is highly compartmentalized [128]. The discovery of a functionally distinct mitochondrial FADS1, alongside alternative splice variants that localize to defined cellular compartments, demonstrates that organelles can synthesize PUFAs in situ [129]. Beyond its traditional cytosolic roles, the subcellular localization of FADS1 is critical for mitochondrial lipid bioenergetics. Specifically, FADS1 localizes to mitochondria-associated ER membranes (MAMs) and the mitochondria directly, where it desaturates fatty acyl chains prior to their incorporation into cardiolipin, the primary dimeric phospholipid of the inner mitochondrial membrane (IMM) [130, 131]. Cardiolipin directly regulates IMM biophysics; its unique conical shape is essential for maintaining cristae architecture, assembling electron transport chain (ETC) super-complexes, regulating mitochondrial fusion, and controlling the threshold for apoptosis [130–132]. The function of cardiolipin depends heavily on its acyl-chain composition, which is continuously remodeled by the transacylase tafazzin and other acyltransferases [130]. By regulating the degree of unsaturation in cardiolipin precursors, FADS1 controls IMM structure, membrane fluidity, and baseline respiratory efficiency [130, 131]. Cancer cells frequently co-opt this localized lipid remodeling. Aberrant FADS1 activity at the mitochondria drives major bioenergetic shifts that support hyperactive tumor respiration while protecting the organelle against apoptosome activation [131]. Highlighting the therapeutic potential of this pathway, targeted FADS1 ablation in renal cell carcinoma triggers an ATF3-driven proteotoxic ER stress response due to unresolved lipotoxicity at the MAM interface. This dual disruption of ER- and mitochondria-localized desaturase activity significantly inhibits tumor growth in both in vitro and in vivo models, establishing the inter-organelle FADS1 network as a strong therapeutic target [133].

Mapping these compartmentalized lipid desaturation networks, particularly at mitochondria-associated membranes (MAMs), will clarify how discrete lipid desaturase activity regulate cell death [134]. Furthermore, resistance to ferroptosis has expanded beyond GPX4 with the discovery of ferroptosis suppressor protein 1 (FSP1), an independent system that utilizes NAD(P)H to regenerate ubiquinol (CoQ10) at the plasma membrane, scavenging lipid peroxyl radicals [135, 136]. Identifying these distinct spatial hubs is critical for generating precision inhibitors.

#### The therapeutic dichotomy: exploiting PUFA overload

While desaturase inhibition remains the primary pharmacological strategy, an unconventional alternative involves the targeted induction of PUFA biosynthesis [48, 137].

Beyond direct FADS-driven PUFA biosynthesis, the pathways governing bulk lipid scavenging, neutral lipid sequestration, and acyl-chain utilization represent alternative therapeutic targets to modulate ferroptosis susceptibility. The composition of the extracellular lipid environment heavily influences this vulnerability. When cancer cells are subjected to severe lipid deprivation, their sensitivity to ferroptosis increases dramatically. The lack of exogenous lipids forces the cells to rely on *de novo* PUFA biosynthesis and rapidly depletes protective lipid droplet reservoirs, shifting labile PUFAs directly into vulnerable membrane phospholipid pools [69]. Conversely, the endocytosis of circulating lipoproteins via surface glycosaminoglycan-dependent receptors protects tumors from ferroptosis [138]. The fatty acid transporter CD36 plays a dual, context-dependent role in this process. In certain cancers, CD36-mediated internalization of pre-oxidized lipids directly primes cells for ferroptosis. In other cancer types, CD36-driven lipid uptake primarily fuels triacylglycerol (TAG) synthesis, expanding the neutral lipid pool and establishing a ferroptosis-resistant state [139, 140]. Intracellularly, this spatial lipid sorting is regulated by lipid storage dynamics. While the DGAT-mediated esterification of labile PUFAs into inert TAGs protects cells from lethal lipid peroxidation, the subsequent breakdown of these lipid droplets via lipophagy replenishes the membrane PUFA pool, rapidly restoring sensitivity to ferroptosis [73, 75]. Consequently, combining canonical ferroptosis inducers (such as RSL3 or Erastin) with CD36 blockade, DGAT inhibition, or ACSL4 activation represents a highly rational therapeutic strategy.

#### Precision modalities: dietary modulation

The cancer cell lipidome is heavily influenced by systemic dietary lipids, which modulate both the SCD1 and FADS catalytic networks [141]. High intake of SFAs expands the intracellular substrate pool for SCD1, driving a feed-forward upregulation of the desaturase via the SREBP1 transcriptional axis. Conversely, dietary supplementation with ω-3 PUFAs attenuates SREBP1-driven lipogenesis and downregulates SCD expression [142]. At the enzymatic level, exogenous ω-3 PUFAs, specifically eicosapentaenoic acid (EPA) and docosahexaenoic acid (DHA), act as competitive substrates for the FADS1/2 enzymes. This competitive displacement shunts long-chain PUFA biosynthesis away from arachidonate (AA)-derived pro-inflammatory eicosanoids, favoring the production of pro-resolving lipid mediators [142]. The impact of these dietary shifts is heavily dependent on host genetics. Single-nucleotide polymorphisms within the FADS1/FADS2 genomic locus determine the catalytic efficiency of exogenous PUFA conversion, meaning the metabolic response to nutritional intervention is fundamentally stratified by patient genotype [143]. In preclinical models, systemic ω-3 PUFA enrichment limits cholesterol-driven tumorigenesis and enhances the antitumor efficacy of immune checkpoint blockade in murine non-small cell lung carcinoma (NSCLC) [144]. Furthermore, marine-derived lipid supplementation prevents compensatory, chemotherapy-induced SCD1 hyperactivation and restores the intracellular EPA pool in tumor-bearing hosts [142]. In contrast, high-fat diets enriched in saturated or monounsaturated fatty acids promote a ferroptosis-resistant state. In lung adenocarcinoma models, these lipid-rich diets repress ACSL4 transcription, significantly reducing sensitivity to ferroptosis [145, 146]. Consequently, exploring the interactions between lipid desaturase inhibitors and dietary modulation represents a promising area for precision oncology.

This compensatory uptake mechanism, whereby cancer cells scavenge exogenous lipids to maintain growth under nutrient-limited or metabolically altered conditions, provides a strong rationale for targeted dietary interventions [147–149]. For instance, tumor cells can scavenge unsaturated fatty acids from the environment, rely on fatty acid receptors like CD36 to fuel metastatic potential, or hijack lipids directly from surrounding adipocytes to support rapid proliferation. By implementing PUFA-enriched clinical diets (e.g., marine-derived omega-3 and omega-6 lipids), clinicians can modulate the lipid species available for tumor uptake, effectively disrupting these survival pathways. Tumor cells readily internalize these circulating PUFAs, thereby increasing their susceptibility to ferroptosis [150]. Conversely, MUFA-rich diets (e.g., olive oils) may counteract therapeutic efficacy, as exogenous MUFAs can act as a systemic rescue mechanism, restoring membrane architecture and promoting resistance to radiation and chemotherapy [151, 152]. The timing and composition of dietary interventions relative to desaturase inhibitor administration are therefore critical pharmacological variables. Enriching tumors with dietary omega-3 PUFAs prior to SCD1 blockade could prime cells for ferroptosis by increasing membrane PUFA content before the protective MUFA synthesis pathway is inhibited. Translating these dietary strategies to the clinic will require evaluating the patient *FADS* genotype, the tumor lipid uptake capacity, and the specific desaturase inhibitor being used.

#### Next-generation nanomedicine and targeted degraders

By exploiting the acidic and hypoxic conditions of the tumor microenvironment (TME), stimuli-responsive nanocarriers offer a promising approach for targeted ferroptosis induction. For example, ultrasmall iron oxide nanoparticles use extracellular acidity to selectively accumulate within tumors. Once internalized by cancer cells, these pH-responsive particles induce ferroptosis and block tumor growth in mouse models, largely sparing healthy tissues from off-target toxicity [153]. Similarly, iron-coordinated metal-organic frameworks (MOFs) have demonstrated anti-tumor efficacy in osteosarcoma and colorectal carcinoma models. Acting as intracellular iron reservoirs, these highly porous structures drive Fenton-mediated ROS generation to effectively induce ferroptosis [154]. Beyond monotherapy, biomimetic vehicles, such as engineered liposomes and exosome-derived nanovesicles, enable the synchronized co-delivery of ferroptosis inducers alongside conventional chemotherapeutics. This combined delivery creates a strong synergistic effect in aggressive triple-negative breast cancer (TNBC) and lung adenocarcinoma models [155]. Despite these promising preclinical results, the clinical translation of pro-ferroptotic nanomedicines remains challenging. Major hurdles include the clearance of metallic nanoparticles by the mononuclear phagocyte system (MPS) and the physical barriers that prevent larger nanoparticles from penetrating deep into dense, desmoplastic tumors. Resolving these biodistribution limits, along with overcoming the challenges of large-scale manufacturing, is a prerequisite for the clinical deployment of these therapies [156, 157].

**Fig. 4:**
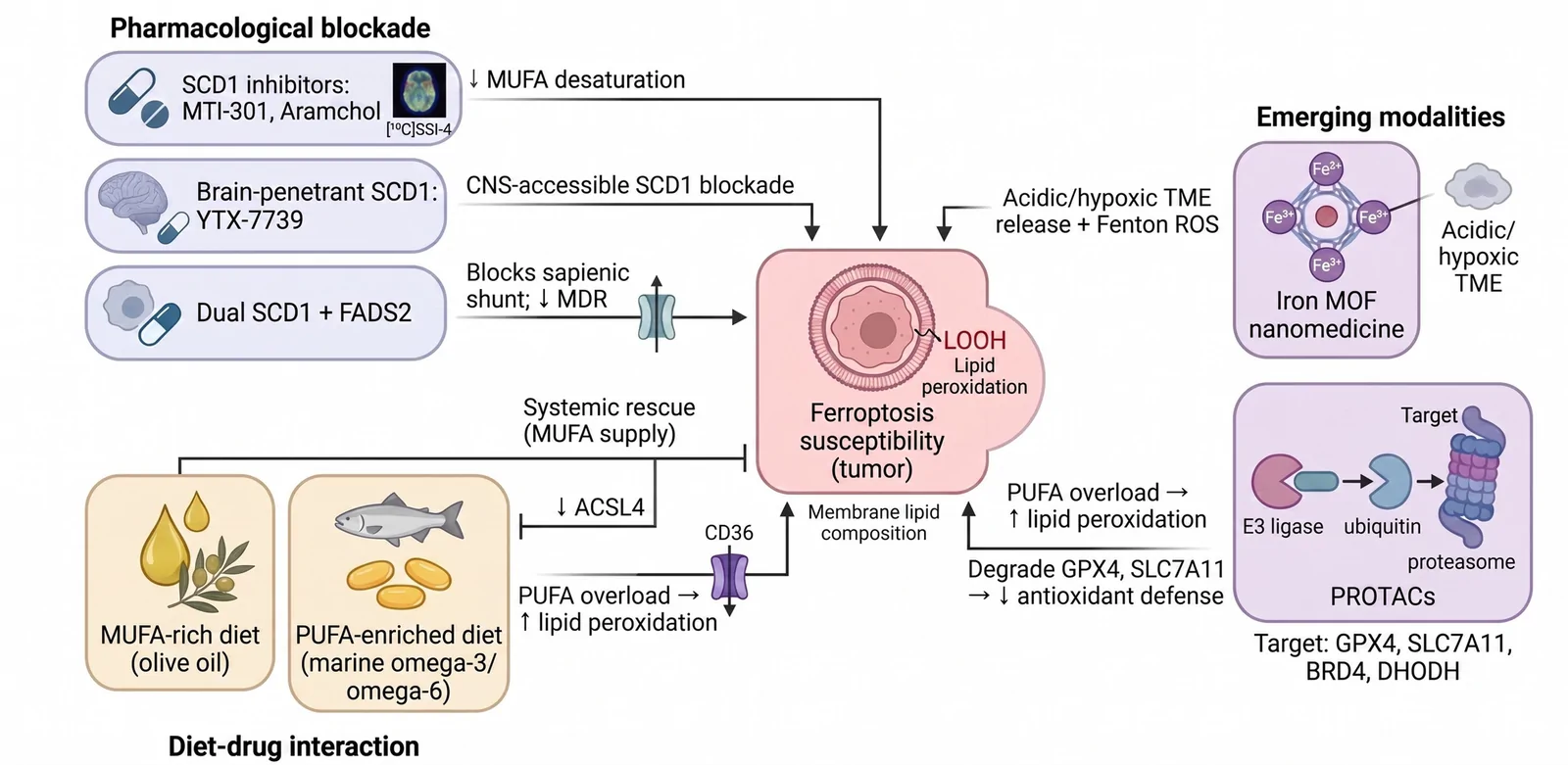
Precision therapeutics and emerging modalities targeting lipid desaturation. Therapeutic strategies aim to shift tumor membrane composition toward PUFA-driven peroxidation while disrupting antioxidant defenses. Systemic (MTI-301, Aramchol) and brain-penetrant (YTX-7739) SCD1 inhibitors reduce cellular MUFA desaturation. The [11 C]SSI-4 PET tracer enables in vivo mapping of SCD1-overexpressing tumors. Dual blockade of SCD1 and FADS2 is required to prevent adaptive lipid rerouting via the sapienic shunt and reverse multidrug resistance. Dietary lipid intake modulates these pharmacological outcomes: MUFA-rich dietary intake provides a systemic source of exogenous MUFAs that can restore membrane MUFA content and suppress ferroptosis, and protect tumor cell membranes by repressing ACSL4, whereas PUFA-enriched clinical diets (marine omega-3\omega-6) drive CD36-mediated PUFA overload, sensitizing the tumor to ferroptosis. Emerging modalities include stimuli-responsive iron metal-organic frameworks (Fe-MOFs) that release Fe2 + in the acidic and hypoxic TME, generating local ROS via the Fenton reaction. Targeted protein degradation using PROTACs leverages the ubiquitin-proteasome pathway to selectively degrade targets like GPX4, SLC7A11, and BRD4, and can be co-delivered with small-molecule DHODH inhibitors to fully neutralize tumor antioxidant defenses

Simultaneously, traditional enzymatic inhibition is being augmented by targeted protein degradation. Key ferroptosis regulators, such as GPX4, have historically been challenging to target with small molecules due to their shallow active sites [158]. Next-generation heterobifunctional molecules, such as proteolysis-targeting chimeras (PROTACs), overcome this structural limitation. These agents recruit the cellular ubiquitin-proteasome system to induce the targeted degradation of GPX4 and SLC7A11 [159, 160]. Pairing localized FADS induction with GPX4-targeted PROTACs represents a promising strategy for precision metabolic oncology. Targeted degradation of GPX4 via PROTACs offers a highly effective alternative to traditional enzymatic inhibitors. For instance, the degrader dGPX4 utilizes the proteasome to completely deplete cellular GPX4, triggering ferroptosis in both cell lines and mouse models with significantly greater efficacy than catalytic inhibitors like RSL3 [161]. To address the poor water solubility and potential off-target risks associated with systemic PROTAC administration, researchers are increasingly turning to nanoparticle encapsulation [162]. This delivery approach also enables advanced combination therapies; for example, co-delivering a PROTAC alongside a DHODH inhibitor within a single nanoplatform produces a strong synergistic ferroptotic response in TNBC models [163, 164]. Similarly, pH-responsive nano-systems delivering BRD4-targeted PROTACs not only induce tumor-specific ferroptosis but also stimulate in situ anti-tumor immunity in melanoma [165]. Despite these advances, PROTAC-based therapies face several critical translational hurdles. These include the pharmacological “hook effect”, where efficacy paradoxically drops at high drug concentrations, the risk of degrading unintended proteins that engage the same E3 ligase, and the physical challenge of achieving high enough intratumoral concentrations to ensure complete target degradation [160]. Addressing these limitations through improved nanocarrier design and tumor-selective activation strategies remains an active area of preclinical investigation (Fig. 4).

## Conclusions

Dysregulation of lipid desaturation, driven by the overexpression of SCD1, FADS1, and FADS2, has emerged as important metabolic vulnerability of cancer. This reliance on desaturated lipids to fuel rapid proliferation and dissemination, maintain membrane plasticity, evade cell death, and resist treatments constitutes a critical metabolic vulnerability across diverse tumor histologies. While exploiting this vulnerability has historically been limited by systemic toxicity, the therapeutic landscape is rapidly advancing. With the recent entry of the first-in-class SCD1 inhibitor (MTI-301) into a phase 1 trial and another SCD1 inhibitor (Aramchol) poised for clinical translation, lipid desaturation-targeted therapies have reached a critical translational milestone. The path forward requires the continuous refinement of these pharmacological strategies: the design of next-generation SCD1 inhibitors that maximize the therapeutic index, the development of novel FADS1/2 inhibitors, the validation of predictive biomarkers for precision patient selection, and the engineering of targeted delivery mechanisms. Future research must systematically evaluate rational combination strategies integrating lipid desaturation-targeted therapies with standard-of-care chemotherapies or immunotherapies. If successfully translated, lipid desaturation-targeted therapy could provide a broadly applicable therapeutic strategy to overcome metabolic plasticity and treatment resistance in highly aggressive malignancies.

## Data Availability

No datasets were generated or analysed during the current study.

## Abbreviations

AA: Arachidonic acid
ABC: ATP-binding cassette
ACSL4: Acyl-CoA synthetase long-chain family member 4
AKT: Protein kinase B
ALA: Alpha-linolenic acid
AML: Acute myeloid leukemia
AMPK: AMP-activated protein kinase
ATF4: Activating transcription factor 4
CCL4: C-C motif chemokine ligand 4
CD8: Cluster of differentiation 8
CD36: Cluster of differentiation 36
cDC1: Conventional type 1 dendritic cell
CoA: Coenzyme A
CoQ10: Coenzyme Q10 (Ubiquinol)
COX: Cyclooxygenase
CSC: Cancer stem cell
CTC: Circulating tumor cell
DGAT: Diacylglycerol acyltransferase
DHA: Docosahexaenoic acid
eIF2-alpha: Eukaryotic translation initiation factor 2-alpha
EMT: Epithelial-mesenchymal transition
EPA: Eicosapentaenoic acid
ER: Endoplasmic reticulum
FABP4: Fatty acid binding protein 4
FADS: Fatty acid desaturase
FASN: Fatty acid synthase
FSP1: Ferroptosis suppressor protein 1
GPCR: G-protein-coupled receptor
GPX4: Glutathione peroxidase 4
IFN-gamma: Interferon-gamma
IRE1-alpha: Inositol-requiring enzyme 1-alpha
LA: Linoleic acid
LC-PUFA: Long-chain polyunsaturated fatty acid
LOX: Lipoxygenase
LPCAT3: Lysophosphatidylcholine acyltransferase 3
LXR: Liver X receptor
MAM: Mitochondria-associated membrane
MOF: Metal-organic framework
mTOR: Mammalian target of rapamycin
MUFA: Monounsaturated fatty acid
NAD(P)H: Nicotinamide adenine dinucleotide phosphate
NADH: Nicotinamide adenine dinucleotide
NASH: Nonalcoholic steatohepatitis
PE: Phosphatidylethanolamine
PERK: Protein kinase RNA-like endoplasmic reticulum kinase
PET: Positron emission tomography
PGE2: Prostaglandin E2
PI3K: Phosphoinositide 3-kinase
PPAR: Peroxisome proliferator-activated receptor
PROTAC: Proteolysis-targeting chimera
PUFA: Polyunsaturated fatty acid
ROS: Reactive oxygen species
RTK: Receptor tyrosine kinase
SCD1: Stearoyl-CoA desaturase 1
SFA: Saturated fatty acid
SLC7A11: Solute carrier family 7 member 11
SPM: Specialized pro-resolving mediator
SREBP: Sterol regulatory element-binding protein
TAM: Tumor-associated macrophage
TKI: Tyrosine kinase inhibitor
TME: Tumor microenvironment
UFA: Unsaturated fatty acid
UPR: Unfolded protein response
YAP: Yes-associated protein
